# Influenza a H1N1 infection complicated with encephalopathy and acute pancreatitis: a case report

**DOI:** 10.1186/s12887-024-04651-z

**Published:** 2024-03-05

**Authors:** Junhao Cui, Wanyu Jia, Peng Li, Xue Zhang, Zheng Li, Chunlan Song

**Affiliations:** 1 Children’s Hospital Affiliated to Zhengzhou University, Henan Children’s Hospital, Zhengzhou Children’s Hospital, No. 1, University South Road, Erqi District, Zhengzhou, Henan Province 450000 China; 2 Beijing Children’s Hospital, Capital Medical University, National Center for Children’s Health, Beijing, 100000 China

**Keywords:** Influenza, H1N1, Encephalopathy, Acute pancreatitis, Complication

## Abstract

This paper reports a case of influenza complicated with influenza associated encephalopathy complicated with acute pancreatitis. This kind of disease is relatively rare, we hope to draw people’s attention to it in order to improve early detection and prognosis.

## Introduction

The variability and unpredictability of influenza virus often leads to the outbreaks of influenza worldwide. During seasonal influenza epidemics, children had a high risk of influenza and susceptibility to severe cases. According to the World Health Organization data, 20–30% of children suffer from seasonal influenza each year worldwide, with approximately 30% developing complications. Influenza has an acute onset and most of them are self-limiting, but some can progress to severe influenza due to complications such as pneumonia. Moreover, a few severe cases progress rapidly and die due to concurrent acute respiratory distress syndrome and/or multiple organ failure, threatening human life and health. Here, we reported a case of influenza A (H1N1) combined with encephalopathy and acute pancreatitis, which has not yet been previously reported. We hope to draw attention to similar diseases and improve their prognosis by identifying them early. An oral informed consent was obtained from the parents of the patient for publication of this case report.

## Case presentation

A 6-year-old girl was admitted to our hospital due to intermittent fever for 11 days, vomiting, coma, and convulsions for 6 days, and mechanical ventilation for 4 days. The child had a history of frequent upper respiratory tract infections and urticaria. The child developed repeated fever (peak of 39.5 °C) after contact with a patient with influenza and tested positive for influenza antigens at a local clinic, then she was treated with oseltamivir phosphate granules, and other symptomatic treatment for five days. Six days ago, the child gradually developed vomiting, lethargy, coma and convulsions. The convulsions were recurrent and last for 2–3 min relieved by sedative drugs each time, manifested as loss of consciousness, trismus, upturned eyes, rigid limbs, shaking, salivation, urinary incontinence. Physical examination showed moist rales in both lungs. The child was then treated with intravenous peramivir for 3 days and immunoglobulin for five days, and was given mechanical ventilation four days ago. The child’s condition did not improve and was transferred to our hospital.

After admission, the throat swab viral nucleic acid detection of influenza A (H1N1) was positive. The chest X-ray showed patchy shadow of increased density can be seen in both lungs. The dynamic EEG showed: (1) more than 60 electro-clinical seizures were monitored; (2) diffuse slow wave; (3) interictal sharp (spike) slow complex periodic discharges; (4) paroxysmal epileptiform wave firing, especially on the right side. Cerebrospinal fluid routine and biochemistry were normal and H1N1 influenza antibody was positive. The cranial MRI showed increased signal intensity on DWI in bilateral hippocampus, medial inferior frontal lobe, bilateral external capsule and cerebral cortex around bilateral lateral fissure, low signal intensity on ADC map, and slightly high signal intensity on T2WI and FLAIR images. Day 6 of admission, the patient had abnormal urine color, which was significantly deeper and darker yellow, and there were no abnormalities in urine routine and color Doppler ultrasound of urinary system and biliary system, myoglobin was normal, rule out urinary tract disease and biliary tract disease, and the lab analysis showed an increased levels of serum amylase (822.0 U/L), serum lipase (126.0 U/L), and urine amylase (1320.0 U/L). The ultrasound showed slightly larger pancreas, and CT showed diffusely large pancreas. The child did not receive treatment with drugs that could easily cause pancreatitis before the onset of pancreatitis, and the patient had no history of allergies and other abnormal adverse reactions, so we ruled out drug-induced pancreatitis. Cystic fibrosis could be ruled out as there was no unusual family history of the child and the chest X-ray did not show the typical signs of lung hyperinflation and bronchial wall thickening. Final diagnosis: (1) severe influenza A (H1N1), (2) influenza-related encephalopathy, (3) acute respiratory failure, (4) pneumonia, (5) acute pancreatitis.

After admission, the child was given a combination of antiviral, anti-infective, cranial hypotension, respiratory support, anti-seizure, intravenous octreotide, and rehydration, along with fasting. Her consciousness was gradually improved, convulsion attack was gradually reduced, spontaneous breathing was restored. The reexamination of serum amylase, urine amylase, serum lipase and pancreatic color ultrasound showed normal. The 18th day of admission, the ventilator was withdrawn and the patient was transferred to the neurology department for continuous treatment. Day 21 of admission, the patient was transferred to the rehabilitation department due to slightly high muscle tension, and was clinically recovered and discharged after nearly 2 months of treatment. After 1 month of discharge, the child was followed up by telephone, and had no convulsive seizures, and motor, language, and intelligence were unremarkable compared with those before illness.

## Discussion

In this case, combining the child’s medical history, clinical manifestations, signs, ancillary examinations and imaging manifestations, the diagnosis of severe influenza A (H1N1), influenza-related encephalopathy, acute respiratory failure, pneumonia and acute pancreatitis was clear. Both influenza-associated encephalopathy (IAE) and acute pancreatitis (AP) are rare and severe extrapulmonary complications after influenza. Fortunately, after nearly 2 months of aggressive medical treatment, the child was discharged with clinical recovery and no serious sequelae remained.

The child had a history of frequent upper respiratory tract infections and urticaria, and although tests related to the child’s immune function did not show significant abnormalities and immunodeficiency could be ruled out, the child was still at high risk for severe influenza. One large study found that less than 10% of flu-infected children had neurological complications such as febrile seizures, encephalopathy, encephalitis, or acute necrotizing encephalopathy [1]. Infection-related encephalopathy refers to central nervous system dysfunction such as convulsion, disturbance of consciousness and increased intracranial pressure, most of which are associated with viral infection [2]. The alteration of number of blood cells and content of protein in the cerebrospinal fluid is usually unremarkable. The cerebrospinal fluid routine and biochemistry of the child in this case were normal, and viral and bacterial encephalitis could be excluded. Combined with the fact that the child appeared to have neurological abnormalities 5 days after the influenza infection, the diagnosis of IAE was made. IAE refers to the clinical manifestations of brain dysfunction syndrome such as fever, convulsion, disturbance of consciousness, and coma in the onset of acute influenza, which easily occur in children. The uncontrollable convulsive seizures of IAE reach 80–90%, whose mortality is up to 37.0% [3]. Since IAE has characteristics of acute onset, rapid progression, poor prognosis, serious consequences, all clinicians should be alert for the development of this disease.

Acute pancreatitis in children is not rare which can occasionally manifest with moderate to severe symptoms. AP had become a common general pediatric condition with an increasing incidence over the past 2 decades. In adult, gallstone disease and alcohol are the 2 leading causes of acute pancreatitis but cases in children have been associated with various etiologies. AP had been associated with several viruses, including mumps virus, measles morbillivirus, Epstein-Barr virus, and coxsackievirus [4]. Several cases of severe influenza A combined with acute pancreatitis have also been reported in the past [5]. It has also been demonstrated in mice that influenza virus can infect the pancreas and cause pancreatic injury [6].Studies had shown that drugs are also the main cause of acute pancreatitis in children. Moreover, cases of drug-induced acute pancreatitis are more severe than those of non-drug-induced [7]. Other causes of pediatric AP include autoimmune disease, trauma, genetics, and anatomical abnormalities. Diagnosis of acute pancreatitis requires the presence of two out of three criteria: (1) abdominal pain suggestive of pancreatitis, (2) serum amylase and/or lipase greater than 3 times the upper limit of normal, (3) and/or imaging tests showing characteristic findings [8]. Children with severe influenza A(H1Nl) present with gastrointestinal symptoms such as nausea, vomiting and abdominal pain, must be promptly tested for blood amylase and urinary amylase, and promptly improve pancreatic ultrasound and pancreatic CT testing, and be alert to the occurrence of AP.

The symptoms of acute pancreatitis presentation may vary by patient age as the signs and symptoms can be more subtle and nonspecific in toddlers relative to older age groups [9]. For this child, due to the state of mechanical ventilation, vomiting, abdominal pain and other symptoms are atypical, the first manifestation of urine color deepening, which also suggests that our children with severe influenza in the presence of deepening of the color of the urine, after the exclusion of urinary and biliary diseases also need to be vigilant for the occurrence of acute pancreatitis. For better of management, depending on whether there are local complications and the presence and duration of organ failure, acute pancreatitis in children is classified as mild, moderate, and severe as in adults. Within this classification, the group also characterized acute recurrent pancreatitis(ARP) as two or more distinct episodes of AP with return to baseline between episodes [10].Roughly 20–40% of children with recurrent AP will progress to chronic pancreatitis in 2 to 5 years [11].Unfortunately, there are no recommended guidelines for monitoring patients with ARP for progression into chronic pancreatitis. Therefore, early recognition and Supportive care with fluids, pain management, and nutrition support are critical. Pediatric patients who received enteral nourishment within the first 24 to 48 h after presentation showed a shorter period of hospitalization when compared to those who did not. So early enteral nutrition is encouraged [12]. Growth inhibitors and their analogs (octreotide) can exert anti-pancreatitis effects by directly inhibiting pancreatic exocrine secretion, and can also combat SIRS [13], and the treatment of this child with octreotide achieved favorable results. For complicated or severe pancreatitis, specialized interventions may be required with endoscopic or drainage procedures [8].

There are few reports of influenza A (H1N1) combined with either IAE or AP. However, there is no report of influenza A (H1N1) combined with both IAE and AP until now. To the best of our knowledge, we here first report a case of influenza A (H1N1) complicated with both IAE and AP at the same time, which will draw attention to similar diseases for early identification and improvement of prognosis.

## Data Availability

The datasets used and/or analysed during the current study available from the corresponding author on reasonable request.

## Abbreviations

IAE: Influenza-associated encephalopathy
AP: Acute pancreatitis
ARP: Acute recurrent pancreatitis
